# End-of-life dementia care: a qualitative study of the experiences and perceptions of minority ethnic and economically disadvantaged groups

**DOI:** 10.1093/ageing/afaf168

**Published:** 2025-06-24

**Authors:** Louise Tomkow, Marie Poole, Efioanwan Damisa, Barbara Hanratty, Faith Tissa, Malcolm Ngouala, Josie Dixon, Maria Karagiannidou, Margaret Ogden, Felicity Dewhurst

**Affiliations:** Humanitarian and Conflict Response Institute, School of Arts, Languages and Cultures, The University of Manchester, Manchester, UK; Ageing and Complex Medicine, Northern Care Alliance NHS Foundation Trust, Salford, Manchester, UK; Population health sciences institute, Newcastle University, Newcastle upon Tyne, UK; Population health and primary care, The University of Manchester, Manchester, UK; Population Health Sciences Institute, Biomedical Research Building Newcastle University, Newcastle upon Tyne NE4 5PL, UK; Population Health Sciences Institute, Newcastle Biomedical Research Building Campus for Ageing , Newcastle upon Tyne NE4 5PL, UK; Population health sciences institute, Newcastle University, Newcastle upon Tyne, UK; Department of Health Policy, London School of Economics and Political Science, PSSRU Houghton Street, London WC2A 2AE, UK; School of Public Policy, The London School of Economics and Political Science, London, UK; Alzheimer’s Society, London, UK; Population health sciences institute, Newcastle University, Newcastle upon Tyne, UK

**Keywords:** end-of-life, dementia, experiences, carers, minority ethnic, qualitative research, older people

## Abstract

**Background:**

Dementia is a leading cause of morbidity and mortality amongst ageing populations. However, palliative care is often poor or non-existent. People from minority ethnic and economically disadvantaged communities are especially likely to miss out. Research exploring how inclusive end-of-life (EOL) care should be provided for people living with dementia (PLWD) is limited and commonly fails to include sufficient representation of people from minority ethnic groups and those living in poverty.

**Aims:**

Our aim was to understand the experiences and perceptions of EOL dementia care amongst underserved groups and investigate how ethnicity and socioeconomic status influence experiences and perceptions of EOL dementia care.

**Methods:**

Ten workshops were held with a total of 29 Experts-by-Experience (EbE). All had professional and/or personal experience of care for people from disadvantaged groups living with dementia in England. The groups explored (i) current evidence gaps, (ii) barriers and facilitators to EOL care for PLWD across different ethnic and socioeconomic groups and (iii) recommendations for care and future research. The discussions were recorded, transcribed and the qualitative data analysed thematically.

**Results:**

Data point to cultural, socioeconomic and systemic barriers to accessing quality EOL care for PLWD. EbE described how there is fear and stigma of talking about dementia and EOL care, and a mistrust of health and care services. Financial concerns were pervasive throughout the data and were described by EbE as significant drivers of inequalities in access to quality care.

**Conclusion:**

EbE from minority ethnic and socioeconomically disadvantaged groups report poor experiences of EOL dementia care. Our data suggests there is a need to improve multiple aspects of care, including access and coordination. Future research should work with underserved communities to co-produce culturally sensitive interventions to address inequalities in dementia EOL care.

## Key Points

- Some minority ethnic and disadvantaged groups reported poor end-of-life dementia care and described mistrust of health and care services.
- Stigma around care homes was described, as was the pressure this can have on family caregivers.
- Trust-building and culturally sensitive communication are crucial. Involving community-based professionals can help.

## Introduction

Dementia has far-reaching health, social and fiscal impacts and is a key priority for healthcare providers and policymakers worldwide [1]. One in two people in high-income countries will be affected by dementia, either by developing the disease or caring for someone with it [2]. Despite dementia being an international leading cause of death, people living with dementia (PLWD) remain under-represented in specialist palliative care services [3]. In the UK, palliative dementia services and associated research are underdeveloped, particularly in comparison with other life-limiting diseases, such as cancer [4, 5]. Where research does exist, it highlights the need to overcome barriers to dementia palliative care provision and access and increase rates of advance care planning [6, 7]. Palliative care is the provision of active, holistic care (control of physical and psychological symptoms and social and spiritual problems) of patients with life-limiting illness and their families by generalist and specialist providers (community and hospital staff who were and were not palliative care specialists). Palliative care includes but is not limited to end-of-life (EOL) care, defined as the holistic care received in the last days to short weeks of life [8]. Studies exploring the experiences of dementia care from the perspective of carers stress the importance of good communication between family and care providers, the need to coordinate care across different settings, and the challenges of recognising dying [9–11]. Many of these studies call for earlier and better EOL discussions and the recognition of dementia as a terminal condition.

Existing research provides insights into the attitudes of caregivers and highlights the gap in palliative care for PLWD; the primary focus is on often-heard populations. Limited attention has been given to the experiences of minoritised groups and those experiencing poverty [12–14]. Numerous calls have been made for the development of culturally appropriate palliative dementia care, and national resources to support this are available from NHS England and Alzheimer’s UK [15–18]. However, there is little qualitative research exploring the perspectives and lived experiences of underserved populations. This is important for several reasons. First, demographic data points to rising prevalence of dementia amongst minority ethnic communities, particularly in Black and Asian populations, where higher rates of chronic health problems increase the risk of developing dementia [19]. Dementia prevalence also has an inverse relationship with socioeconomic status, with higher rates in those with the lowest income [20]. Second, people from minority ethnic groups and those experiencing poverty are more likely to face barriers to accessing appropriate and timely healthcare. For people from minoritised ethnicities, barriers to palliative care can include stigma, mistrust of providers and experiences of racism and discrimination within healthcare settings [14, 21, 22]. Cultural factors, such as the derogatory terminology for dementia in some communities, exacerbate these challenges and can lead to delayed presentations for treatment and care [14, 23]. Research also shows that people from minority ethnic groups access healthcare later in the dementia trajectory and are less likely to access medication or 24-hour care [24]. Third, the underrepresentation of marginalised groups in research means that needs are not well understood and therefore remain overlooked in the way that palliative dementia care services are designed and delivered [19]. This study seeks to understand the experiences and perceptions of palliative dementia care amongst under-served groups and investigate how ethnicity and socioeconomic status influence dementia care at the EOL.

## Methods

### Workshop design and objectives

A series of workshops was designed to engage diverse stakeholders. The objectives were to (i) gather insights on the experiences of people from disadvantaged groups to identify their perspectives on key issues in dementia care at the EOL and (ii) explore how ethnicity and socioeconomic status might influence palliative dementia care.

### Patient and public involvement and engagement and research team positionality

The research team included two funded co-researchers with lived experience of both dementia and minoritisation. This included being from, directly related to or working with, those from; minoritised ethnic, socioeconomically deprived, urban and rural communities and those with refugee and asylum seeker status. They were able to offer key insights into intersectional aspects of disadvantage in palliative dementia care (MN and MO). The co-researchers acted as co-production partners and were embedded in the research team, attending weekly meetings and contributing to study design, recruitment, data collection, data analysis and dissemination. Co-production methods are underpinned by the principle that those affected by the issues under study are best situated to play a central role in the design and delivery of research, working together with the research team to share power, responsibilities and the generation of knowledge [25]. The remaining members of the research team, all female, were academics and clinical academics from three UK higher education institutions, whose experience ranged from postdoctoral early career researcher to professor. All clinical academics had professional experience in dementia and EOL care. Except for FT, whose doctoral research focused on ethnicity and identity, all academics had experience researching dementia and EOLC.

### Participant recruitment and selection

Experts by Experience (EbE) who had either professional and or personal lived experience of EOL care for PLWD were recruited between April and July 2023. Purposive snowball sampling was used, capitalising on existing networks within the research team, including voluntary and community sector enterprises, religious groups and health and social care, dementia and palliative care services. Relationships were not established between researcher and participant before the study. Invitations were extended via email and in-person. Snowballing enabled recruits to signpost to additional EbEs. EbE were capacitous individuals aged ≥18 years who were understood to be ‘information rich’, knowledgeable and interested in dementia and EOL care, either from a service user (personal) or professional perspective. Purposive sampling ensured a broad representation of diversity in ethnicity and socioeconomic status and ensured a balanced inclusion of people with both personal and professional experience [26]. Consideration of potential participants’ lived experiences, including of poverty, bereavement and ethnicity, was approached with sensitivity. To understand their experiences, we asked potential participants to provide a short, informal written or oral biography of their circumstances and interest in the project. A subjective judgement was then made by FT, LT, MP and FD based on the issues they highlighted. No participants were excluded through this process.

### Workshop format and facilitation

Ten workshops were conducted between July 2023 and February 2024 with each lasting 2 hours. Two workshops were held in person and eight online via Zoom. The in-person workshops were conducted in a deprived suburb in Greater Manchester to accommodate participants’ preferences, logistical needs and digital literacy/access. One individual interview was conducted online to allow an information-rich EbE, who was unable to attend online, to take part.

The workshops were facilitated by two or three members of the research team, who ensured that discussions remained focused and inclusive. Supplementary Appendix 1 outlines the workshop format. All authors facilitated at least one group. Each workshop followed a structured format, beginning with an introductory session to outline the workshop’s objectives, introduce the research team, including their credentials and interest in dementia in EOL care, recap previous sessions and establish guiding principles for discussions. Following this, the workshop then opened for discussion, following a semi-structured format where facilitators led the group through a series of open-ended questions and prompts designed to elicit diverse perspectives on dementia palliative care. The workshops concluded with a brief synthesis session, where key themes and potential action points were identified collectively.

The workshops were conducted in three stages. The first explored EbE experiences of palliative dementia care. To prompt discussion, findings from a scoping review of the current literature (in press) were shared in the form of a one-page plain English summary. This was distributed with EbEs prior to the workshop. The second set of workshops facilitated discussions about solutions to key issues that emerged from the initial workshops. The final workshops revisited issues that arose as important in earlier sessions and supported the production of study outputs. All 29 participants were invited to attend each stage, though attendance was tailored to individuals’ abilities and preferences, hence the in-person workshops. The initial workshops separated participants with personal experience from those with professional experience to promote open discussions and address potential power imbalances. This approach aimed to create a safe space and tailor the language and tone to each group’s needs. However, many participants had both types of experience, leading to common themes across both groups. As a result, the final round of workshops adopted a mixed-group format to reflect the shared insights and experiences of all participants.

To support discussions, one-page ‘what you told us’ plain English summaries of key findings from earlier sessions were circulated to EbE before the second and third series to provide a summary overview of key findings from the previous phase. This ‘sense-making’ approach allowed EbE to reflect on and adjust their views and observe in real time how their views influenced the study findings [27]. During the online workshops, EbE were invited to use Miro boards—an interactive online whiteboard [28]. One member of the research team annotated key themes emerging during the discussion in real-time, meaning the Miro also served as a form of visual minute-keeping. Miro boards allow more than one participant to contribute simultaneously, provide a platform to share views without verbalising and facilitate the creation of a visual summary record of the conversation. The ‘what you told us’ documents were generated through a rapid analysis of researchers notes and the Miro boards [29].

### Data collection

Data was collected using a combination of qualitative methods. These included audio recordings of the sessions, detailed facilitator notes and Miro boards. Audio recordings were transcribed verbatim, and written materials were digitised for analysis. Facilitator notes were used to supplement the transcriptions, providing context and highlighting non-verbal communication or group dynamics that were not captured in the recordings.

### Data analysis

The data were analysed using thematic analysis, following the steps outlined by Braun and Clarke [30]. A phenomenological theoretical orientation was used to focus on lived experiences and emphasise meaning-making [31]. The process began with familiarisation with the data, where the research team reviewed the transcriptions and written materials multiple times. Initial codes were generated based on recurring concepts, ideas or issues identified in the discussions. These codes were then organised into broader themes that captured the essence of the stakeholders’ perspectives.

All authors participated in the data analysis process, with peer support given to co-researchers. Multiple researchers independently coded a subset of the data and then compared their findings to refine the coding framework. To ensure analytical rigour, online and in-person data meetings were conducted where three team members met to discuss and their analyses. This ensured we made time to compare and discuss our different perspectives and challenge our assumptions, thus allowing a range of interpretations of the data to be considered. Disagreements were resolved through discussion, and the final set of themes was agreed upon by the entire research team. The diverse positionality of the research team, clinicians, qualitative researchers from various disciplinary and personal backgrounds, and lay co-researchers with lived experience, enriched the analysis of the data. Clinicians on the research team provided practical insights into the challenges of EOL dementia care, ensuring the coding framework captured relevant clinical themes. Qualitative researchers contributed their expertise in thematic analysis, maintaining methodological rigour and shaping data interpretation with theoretical perspectives. Lay co-researchers with lived experience grounded the analysis in the realities of those affected by EOL dementia care, uncovering themes that may have been overlooked by researchers alone. For example, the theme of mistrust of formal healthcare was identified by MN.

### Ethical considerations

Ethical approval for the study was obtained from Newcastle University (Reference: 02560-31564). Informed consent was obtained from all participants prior to the workshops, with assurances that their contributions would be anonymised in any publications or reports. Participants were also informed of their right to withdraw from the study at any point without penalty.

Confidentiality was maintained throughout the research process, with all data stored securely and accessible only to the research team. Any identifying information was removed from the transcriptions and reports to protect participants’ anonymity. In line with NIHR guidance, EbE were offered a £25 voucher for their contribution to each workshop.

## Results

Twenty-nine EbEs from diverse backgrounds participated in the workshops (Table 1). All had either personal or professional experiences of EOL care for PLWD in England. Many EbE with professional experience, such as working in dementia care, also had personal experience, such as caring for a relative with dementia at the EOL. During the workshops, it was not always possible to determine whether the person was drawing on their professional or personal experiences when speaking. Often, narratives of personal and professional were interwoven.

**Table 1:** Self-reported demographic characteristics of 29 experts-by-experience

**Ethnicity**	Asian 11	Black 5	White 13		
**Religious Affiliation**	Muslim 6	Jewish 3	Christianity 2	No Stated Religious Affiliation 18	
**Personal and/or professional experience**	Both 14	Personal 5		Professional 10	
**Profession**	Doctor 1	Nurse 4	Paid carer 4	Social Worker 3	Voluntary community organisation 6
**Personal**	Family carer -bereaved 7	Family carer – current 4			
**Residential**	Experienced homelessness 2	Refugee 1	Asylum seeker 1		
**Organisation**	Palliative care 4	Geriatrics 2	Primary care 2	Care Homes 4	VSCO 6
**Gender**	Male 10	Female 19			

The results are presented across the following themes: fear and misunderstanding about dementia and palliative care, cultural influences, financial constraints and inadequate funding.

Codes and subthemes are presented in Table 2. Although the findings are presented thematically, the narratives developed across and between the three workshops. In the first workshop, participants primarily focused on introductions, outlining their personal and professional experiences and identifying key barriers to quality EOL care, such as fear, stigma and financial constraints. In the second workshop, guided by the ‘what you told us’ summary and the scoping review findings, the discussion revisited these barriers and moved towards exploring potential solutions. Solutions included policy changes, community-led initiatives and culturally sensitive care models. The final workshops revisited these solutions and focused on summarising the key messages of the workshops to aid the dissemination of the study’s findings. Throughout the three-workshop series, EbE drew on their personal and professional experiences to justify and provide evidence for their ideas and suggestions. These three co-themes, therefore, cut across these experiences.

**Table 2:** Sub-themes and codes from qualitative data analysis

Main theme	Sub-theme	Code
**1. Fear and Misunderstanding about Dementia and Palliative Care**	- Misunderstanding of dementia as a palliative condition	- Lack of awareness that dementia worsens over time and limits life expectancy.—Dementia is not seen as a condition that requires EOL planning.
	- Reluctance to discuss EOL planning	- Resistance to asking questions about EOL due to not viewing dementia as a terminal condition.
	- Cultural stigma around discussing EOL	- In some faiths and cultures, discussing death is believed to ‘invite it in’.—Debate on whether this stigma is present only in minority ethnic communities or also amongst white British.
	- Mistrust towards medical care at EOL	- Suspicion towards medical interventions, e.g. injections, perceived as ‘injections of death’.—Concerns about excessive pain relief being administered without explicit consent, akin to euthanasia.
	- Personal experiences influencing perceptions	- Stories of family members and loved ones perceived to have received inadequate or harmful care.
**2. Cultural and Religious Influences**	- Stigma associated with care homes	- Negative perception of placing family members in care homes, seen as a lack of care or selfishness.
	- Cultural norms influencing care decisions	- Preference for keeping care within the family due to cultural and social expectations.
	- Religious beliefs shaping EOL care preferences	- Faith-based practices providing comfort and support, e.g. faith-specific care homes.—Challenges due to funding issues for culturally specific care settings.
	- Lack of progress in culturally sensitive care services	- Perception of minimal change or improvement in care services for minority ethnic communities over time.
	- Dual challenge of information overload and limited guidance	- Families are overwhelmed with information at the time of diagnosis but lack guidance during EOL phase.
**3. Financial Constraints and Inadequate Funding**	- Economic barriers to accessing quality care	- Financial burdens of social care, e.g. ‘top-up’ fees for care homes, funeral costs.—Lack of transparency regarding care costs.
	- Insufficient support structures for carers	- Need for better remuneration, benefits, respite care and support groups for family caregivers.
	- Geographical disparities in resource distribution	- Uneven access to specialised dementia nursing care based on location, e.g. lack of Admiral Nurses in certain areas.
	- Impact of material deprivation on care quality	- Disparities in care quality between wealthier and poorer areas.—Inadequate or non-existent care in prisons and hostels.
	- Advocacy and equitable access to care services	- Need for special attention to ensure fair access to care services, particularly for underserved communities.

### Fear and misunderstanding about dementia and palliative care

EbEs described how dementia is not well understood as a condition that worsens over time and limits life expectancy. As one dementia charity worker described: ‘I can understand the resistance to asking questions about end-of-life planning with dementia because I think a lot of people don’t see it as a palliative condition’. EbEs agreed that these misconceptions could prevent timely conversations about EOL care and planning, leaving individuals and families unprepared for the later stages of the illness. Some highlighted the dual challenge faced by families, who often encounter overwhelming information at the time of diagnosis, juxtaposed with limited guidance and support during the EOL phase. As the disease progresses, this initial information ‘overload’ (EbE 12) often fails to translate into practical support during the often difficult EOL phase.

There was much general and some explicit (EbE 3,6,10) discussion of ‘cultural stigma’ around discussing dementia and EOL care, rooted in beliefs that talking about death invites misfortune. One participant described how some communities fear ‘inviting it in’ by discussing death (EbE 27), highlighting cultural barriers to open dialogue that could aid EOL planning. However, debate arose about whether this reluctance is unique to minority ethnic groups, as several white British EbEs also reported similar fears. EbEs agreed that there was a broader societal unease with mortality and discussed the need for interventions that address both universal and culturally specific factors to foster open discussions about dementia and EOL care.

Misunderstandings surrounding palliative and EOL care emerged from the data analysis and were described by EbEs as pervasive. Several individuals expressed mistrust or suspicion towards medical care for dementia at the EOL.

“In our Asian community, they believe, you know when you come in and give them that injection, and that injection is supposed to ease them a little bit, right? … But then our people think, ‘That’s it. They are giving you the injection of death.’ I remember with my grandad they gave him that injection and literally within 24 hours… he passed away.”

–EbE 5, Community health worker with personal experience

“We found out through this girl who worked in health care that they’d basically given him an overdose of pain relief to end his life. So they’ve killed him. Euthanasia, whatever you want to call it. Prison authorities wouldn’t agree, saying it didn’t happen. They had given him pain relief when he just passed in the night. After talking to lots of people who’ve been in for years and years and years, that seems to be the sort of thing they do in prison.”

–EbE 12, Community worker with personal experience of being incarcerated

In both cases, these perceptions are rooted in personal lived experiences. For a community worker from the Asian community, an injection intended to provide comfort or relief was interpreted as hastening death. Similarly, a stakeholder with personal experience in the prison system suggested that excessive pain relief might be administered to hasten the passing of inmates, potentially without their explicit consent. The confounding of palliative care with euthanasia highlights how constrained communication and opaque decision-making processes, compounded by mistrust towards healthcare providers, can deepen misunderstandings about palliative care practices and their intent.

### Cultural influences

Cultural norms appeared to influence perceptions of dementia and the decisions surrounding care within minority ethnic communities. A recurring theme across cultures was the stigma associated with placing a family member in a care home. During an in-person workshop with members of the South Asian community, one stakeholder became upset describing the strain experienced through caring for her father-in-law with dementia. Another member of the group, a community worker, described how, despite this strain, the community was not necessarily accepting of care homes:

“Care homes, it’s kind of looked down upon in our communities. For example, now if she were to put her father-in-law into a care home, she would get slaughtered… her husband and she would absolutely get slaughtered, thinking, ‘They are selfish. They don’t care about the father-in-law. They are disgusting.’”

–EbE 6, Community charity worker

The stigma associated with both the diagnosis of dementia and seeking care outside the family unit was also described amongst members of the Chinese community:

“You don't talk about [dementia]. It is something personal and you keep it to the family. And it is even worse—it was sometimes used as a—not swearing—but insult. And the care is merely down to family members. And you will be seen as not being a good family member or not caring about your parents if you send them to a care home. It’s like something to be looked down upon. So, I think, no matter how hard it is, the family members will insist they keep the patient around. That will cause problems and pressure within the family as well.”

–EbE 10, Community worker with personal experience

Faith played a crucial role in shaping EOL care preferences for some EbE. Faith-based practices were described as a source of comfort and support, particularly in culturally specific care settings. Much discussion focused on faith-based care homes, with people sharing experiences of excellent EOL care at specialist homes for Jewish and Hindu PLWD. Such facilities were praised for their ability to integrate culturally appropriate rituals, dietary practices and religious observances into care, which contributed significantly to the well-being and dignity of residents towards EOL. However, EbEs described the availability of such care as contingent on insecure funding, as highlighted by a participant who lamented the closure of a faith-specific care home due to financial constraints: ‘They had a faith-specific care home for people with dementia that are Hindu… But then they lost their funding’. (EbE 3).

EbEs called for comprehensive improvements in culturally sensitive care services, noting that systemic inertia has hindered progress. Despite growing awareness of the importance of cultural and faith-based considerations in dementia care, substantive changes remain elusive. This stagnation was powerfully articulated by a social worker, who reflected on the lack of meaningful progress over the past decade.

“I wrote a dissertation in 2012 on the experiences of people from black and ethnic communities with regards to dementia. It hasn’t changed. It hasn’t changed 11 years on.”

–EbE 26, Social worker

### Financial constraints and inadequate funding

Economic factors were identified as a significant barrier to accessing quality dementia care at the EOL. Many participants described the financial burdens associated with social care, such as unexpected ‘top-up’ fees for care homes and the costs of funerals. One social worker expressed concern about the lack of clear communication regarding these costs, stating, ‘Top-ups, for example… it’s come to about £5,000, and in six months they have to go and end up paying it’. (EbE 3). This lack of transparency exacerbates the financial strain on already financially vulnerable families, who EbEs described as frequently unprepared for the costs associated with care at the EOL. EbEs highlighted the financial barriers impeding access to care, with financial assessments for care said to ‘put people off accessing care, including for dementia’ (EbE 26). Though inadequate funding support can be seen across England, this disproportionately impacts people from poorer socioeconomic backgrounds, who already struggle to meet rising costs of living [32, 33].

EbEs collectively underscored the necessity of augmenting support structures for both informal family caregivers and formal care providers. They advocated for improved remuneration for professional carers and emphasised the need for appropriate benefits, respite care and support groups for family caregivers.

“We know how carers aren’t paid particularly well, and some people are carers and they are not paid at all. They face a lot of financial barriers to try to look after their relatives or whoever it happens to be. I think (increasing carers’ wages) would make a big difference to the quality, directly or indirectly, of the support that somebody has and the quality of their life.”

–EbE 15, Personal experience of caring for PLWD

The uneven geographical distribution of public resources further compounds these financial challenges. In some areas, essential services like specialised dementia nursing care are severely underfunded or entirely absent. A participant highlighted this disparity, noting, ‘In [City], we don’t even have an Admiral Nurse… [City] is really underfunded with dementia support’. (EbE 11). This underfunding disproportionately affects underserved communities and exacerbates existing regional inequalities. EbEs described how, as a result, charitable services attempt to fill the gap.

EbEs with professional experience reflected on the difference that material deprivation makes to patients’ experiences of dying with dementia and shared stories of how they had witnessed people from wealthier areas receiving ‘better’ dementia and palliative care. EbEs also described the detrimental effects of extreme poverty on the quality of dementia care, particularly in settings with high representation of those who are more financially vulnerable, such as prisons and hostels, where care was often viewed as inadequate or non-existent. One participant reflected on their own experience in the prison system, stating,

“You don't get end-of-life care. You don't get any dementia care, mental health services in prison. It's pretty dire at the best of times.”

–EbE 12, Community worker with personal experience of being incarcerated

Another, who had experience living in a homeless shelter where PLWD were being housed, reflected:

“Are people with dementia at the end of life really ideally suited to be left in a hostel, amongst, you know, people of multiple age groups from quite young to quite old? To me, it was completely not the right environment. Apart from the odd one or two occasions, I don't think anybody actually came out to see them, though, in terms of health visitors or any type of assistance.”

–EbE 15, Community worker with personal experience of living in a hostel for unhoused people

EbEs agreed that these scenarios warranted special attention to ensure advocacy and equitable access to care services.

## Discussion

Although the current literature outlines inequalities in both dementia care and palliative care, there is a lack of research specifically exploring the intersection of these fields, particularly regarding the experiences of people from underserved communities in England [34]. In this study, minority ethnic groups and people with experience of material disadvantage reported poor experiences of multiple aspects of palliative and EOL dementia care.

Several studies suggest minority ethnic groups face barriers to accessing health and social care in the UK [35–38]. A particularly dominant theme through our data was the fear and mistrust of formal health and care services amongst some minority ethnic communities. This has been described by others and has gained increased attention since the COVID-19 pandemic, which saw lower vaccine uptake amongst minority ethnic groups due to experiences of discrimination in healthcare and resultant mistrust [14, 37]. Our study develops this literature by identifying mistrust of palliative care, e.g. where pain relief at the EOL may be misconstrued as an attempt to hasten death rather than to alleviate pain. This belief, observed across both some minority ethnic groups and people who had been incarcerated, stemmed from lived experiences and community narratives that perceive medical interventions as potentially harmful. Our research develops understanding and adds to the literature outlining the inequities faced by this population in palliative care provision [39, 40]. We do so in a way that is relevant to clinical practice. For practitioners, findings emphasise the importance of clear and compassionate communication about the intentions and rationale behind medical treatments at EOL.

During several of the workshops, EbE discussed whether the issues identified were unique to minority ethnic groups and those living in poverty or were universal across the whole population. One example is the stigma surrounding care homes. Existing literature points to widespread stigma around the care home setting but suggests that this is especially pronounced amongst some minority ethnic groups, who view care homes and hospitals as a last resort [11, 41, 42]. The discussions in our workshops echoed this but added important social and cultural granularity. EbEs from South Asian and Chinese communities described the social judgement and criticism faced by families who utilise care homes. In these discussions, many shared first-hand accounts, revealing the pressure and isolation this can place on family carers. These testimonies begin to paint a more nuanced picture of how this stigma manifests, highlighting how traditional family roles and expectations can influence perceptions of institutional care and how and why barriers to care exist and persist.

We also share examples of good practice; EbEs described how faith-specific care homes provided supportive and individualised EOL care. Therefore, our data suggest that, although the issues described reflect broader systemic challenges within healthcare systems and society at large, issues such as stigma can manifest uniquely within different communities, thus warranting specific attention.

In many cases, our workshops touched on issues already described in the dementia literature but suggested how and why these issues might manifest and be experienced differentially by marginalised groups. EbE self-reported a lack of awareness of the nature of dementia as a life-limiting condition, and some told of how speaking about death and dying was frowned upon within communities for fear of expediting it. Lack of prognostic awareness amongst caregivers has been described elsewhere [43], with calls to increase public awareness and discourse around dementia as a life-limiting condition. Our workshops reveal the limitations of a one-size-fits-all approach to this and show that cultural sensitivity is needed when talking about death and dying. Data also adds to the evidence base around unequal and fragmented service provision [44, 45]. In some cases, we heard of geographical areas where publicly funded dementia support services were believed not to exist. The issue of dementia in prison has received an increase in attention from charities and academics in recent years [46, 47]. The accounts of absent and poor EOL care in hostels and prisons in our research suggest that there is much work to do to improve access to services for this group. We also point to regional inequalities across England and suggest that marginalised communities may need additional help navigating complex systems, including language and financial support [33].

These findings are significant for several reasons. First, they go some way to explaining the underrepresentation of people from minoritised groups in specialist palliative care services and point to where work is needed to improve service equity. Second, they show how complex and difficult-to-access support systems and funding impact those already marginalised and disadvantaged disproportionately. As a result, minoritised communities rely on information about dementia from their peers or online before seeking out professional services, sources that may be vulnerable to misinformation. Lastly, they highlight the importance of research that puts marginalised people with lived experience at the centre. EbE were aware that certain populations had not been considered in dementia research about palliative care, including prison populations and PLWD without family.

### Limitations

The study has several limitations. Although the EbE represented a range of backgrounds, experiences and geographical locations in England, not all ethnicities and experiences of economic challenges took part in this study. Our broad approach provided insight into diverse experiences but may limit transferability. We also recognise that we were unable to consider all potentially relevant factors (including gender, sexuality, migration, Indices of Multiple Deprivation band, educational background, access to digital technology and digital literacy levels) and how they intersect with ethnicity and socioeconomic position. Whilst we did not explicitly collect data on rural/urban spread, the available information points to a concentration of participants from urban settings. Reflecting on diversity within the research team, we acknowledge our own experiences and biases from our cultural and ethnic heritage and how this positionality impacts the research. None of the EbE discussed assisted dying, and we, the research team, did not facilitate discussion on the topic. Nevertheless, this is an important area for future research.

We carefully considered the ethical implications of the workshops, fully consented all EbE, and ensured they were clear about how we would maintain confidentiality beyond the group setting. We are also mindful of the power imbalances between researchers and community members, and between EbE, and how this might have affected the equity of the collaboration. We balanced insights from lay co-researchers with academic perspectives in the project design and data interpretation. There is also potential for bias. Involving community members deeply in the research can make our findings subjective, which can influence the transferability of the themes we developed. Nevertheless, given that the under-served perspective was specifically what we were interested in, we felt that the method’s way of fostering engagement and promoting policy action outweighed its challenges, making it valuable for applied health research.

## Conclusion

EbE from minority ethnic groups and those living in poverty report poor experiences of EOL dementia care. Our data points to mistrust of palliative care within some minority ethnic communities. This, coupled with the stigma surrounding institutional care and a lack of awareness about dementia as a life-limiting condition, underscores significant barriers to accessing equitable EOL care. Our data suggests there is a need to improve multiple aspects of care, including access and coordination.

This study identifies several areas for development for health practice, policy and research. Previous research has highlighted the underrepresentation of people from minoritised groups in EOL care and dementia services. The effort is needed on the part of healthcare providers to build trust with communities to allay some of the fears detailed in this study; EbE suggested partnerships working with community leaders are needed. Finally, we identified several areas for future research, including the role of gender in caregiving at EOL, the issues around advance care planning with minority ethnic groups and the care of incarcerated people and those living in shelters with dementia. Future research should work with underserved communities to co-produce culturally sensitive interventions to address inequalities in dementia EOL care.

## Supplementary Material

Supplementary_materials_afaf168
