# Supplementary material for: End-of-life dementia care: a qualitative study of the experiences and perceptions of minority ethnic and economically disadvantaged groups
Source: Age Ageing. 2025 Jun 24;54(6):afaf168. doi: 10.1093/ageing/afaf168 (PMC12206086; doi:10.1093/ageing/afaf168)
Supplement: Supplementary_materials_afaf168 [file supplementary_materials_afaf168.docx]

# **End-of-life dementia care: A qualitative study of the experiences and perceptions of minority ethnic and economically disadvantaged groups**

**Appendix 1. Workshop outline and topic guide**

All workshops in the project were designed to engage stakeholders in meaningful discussions around issues related to dementia care, particularly end-of-life care, and experiences within minoritised communities. The sessions are structured to foster collaboration, gather input on key research areas, and explore solutions for improving care. The workshops were conducted in a series of three stages, with each stage building upon the previous one.

**Workshop Structure:**

1. **Introduction:** Each workshop begins with a brief introduction to the session's aims and objectives. The facilitators introduce themselves and explain the ground rules for the session, including the importance of respectful dialogue, the Chatham House Rule, and the use of Zoom's features (e.g., hand-raise, chat). Facilitators may also remind participants about the availability of a **Miro** board for further contributions. Participants are reminded of consent protocols.
2. **Project Background and Research Findings:** A key component of each workshop was the presentation of the project’s background and previous research findings. This provides context for the discussion, allowing participants to reflect on prior findings while considering their own experiences and perspectives. Facilitators shared a brief overview of the main points and discuss the findings in relation to the workshop’s focus.
3. **Group discussion:** Each session includes a group discussion designed to facilitate discussion and explore emerging themes. Facilitators present specific prompts related to the findings and the challenges of end-of-life dementia care. Participants are invited to share their thoughts, insights, and recommendations based on their personal and professional experiences. The facilitators guide the discussion and ensure that all voices are heard.
   1. Workshop 1, after research team presented scoping review:
      - Which of these findings/areas do you think are most important/relevant to end-of-life dementia care?
      - Is there anything missing from the findings or discussion?
      - What questions might we want to ask in future studies?
   2. Workshop 2, after recapping barriers discussed in workshop 1
      - How can we improve services?
      - How can awareness and education around dementia, dying with dementia, and end-of-life care be enhanced?
      - How can fear and mistrust of dementia services be overcome?
   3. Workshop 3, after recapping workshops 1 and 2 and discussing planned outputs
      - What do you think of the planned study outputs, with time to develop
      - What do you think are the most important things we have discussed in this project
      - Any other reflections on the project process
4. **Wrap-up:** The session concludes with a summary of the key themes discussed and any next steps. Participants are thanked for their contributions, and additional feedback mechanisms, such as follow-up emails or informal discussions, are introduced.
